# Colosplenic Fistula Following Splenic Embolization in a Sickle Cell Disease Patient

**DOI:** 10.7759/cureus.61011

**Published:** 2024-05-24

**Authors:** Abdullah Al Jabri, Safiya Al Masrouri, Humaid Al Adawi, Hani Al Qadhi

**Affiliations:** 1 Surgery, Sultan Qaboos University Hospital, Muscat, OMN

**Keywords:** case report, splenic embolization, splenic abscess, sickle cell disease, colosplenic fistula

## Abstract

This case report details a rare instance of a colosplenic fistula following splenic embolization in a patient with sickle cell disease. The patient, a 29-year-old female, presented with symptoms of left hypochondrial pain and fever. Imaging revealed a splenic abscess characterized by an air-fluid level. Intraoperative observations disclosed that the spleen was entirely replaced by an abscess cavity, with the presence of colosplenic fistulae. A splenectomy and colonic resection were performed. This report highlights a rare complication that occurred a long time after splenic embolization, underscoring the need for a high level of suspicion to prevent serious complications.

## Introduction

Colosplenic fistulae, though rare, are serious complications often resulting from underlying severe medical conditions or postsurgical interventions. They are most frequently associated with malignancies such as diffuse large B-cell lymphoma or complications arising from perforated colonic non-Hodgkin’s lymphoma. They can also develop from metastatic deposits in the spleen [1,2]. Cases of colosplenic fistulae post-interleukin-2 therapy, which is commonly used in treating cancers due to its strong immunomodulatory effects, have also been documented [3,4]. While the incidence of splenic abscesses post-embolization is relatively low, they rarely progress to colosplenic fistulae [5]. This report discusses a unique case of colosplenic fistula emanating from a chronic splenic abscess years after initial embolization for sickle cell disease (SCD).

## Case presentation

A 29-year-old female with a history of SCD presented with a two-month history of intermittent fever, chills, profuse sweating, and significant weight loss. She described a sharp, stabbing sensation on the left side of her abdomen and absent respiratory or urinary symptoms. Eighteen years ago, she underwent partial splenic embolization for hypersplenism at an outside hospital. This procedure led to the development of an abscess, which was treated with antibiotics and drainage. However, there is no clear documentation on whether the abscess was fully resolved afterward. Her medical history also included multiple hospital admissions for acute chest syndrome requiring exchange transfusions and bilateral hip avascular necrosis. She was allergic to piperacillin/tazobactam.

During the physical examination, she exhibited pallor, tachycardia, and tenderness over the left hypochondrium. Laboratory results indicated a hemoglobin level of 8.2 g/dL (11.0-14.5 g/dL), a white blood cell count of 24 × 10^9^/L (2.4-9.5 × 10^9^/L), and neutrophils at 13.8 × 10^9^/L (1.0-4.8 × 10^9^/L), with a C-reactive protein level of 149 mg/L (0-5 mg/L).

**Figure 1:**
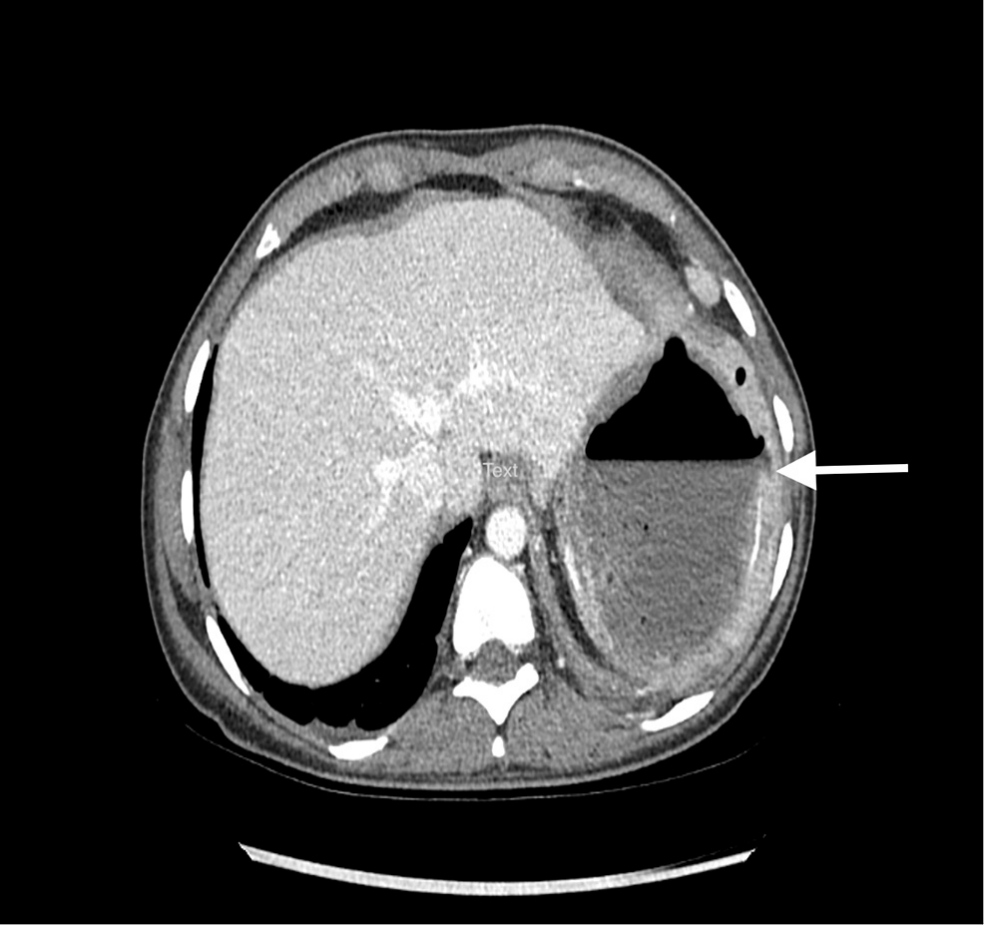
CT scan of the abdomen showing a splenic abscess with an air-fluid level (white arrow).

**Figure 2:**
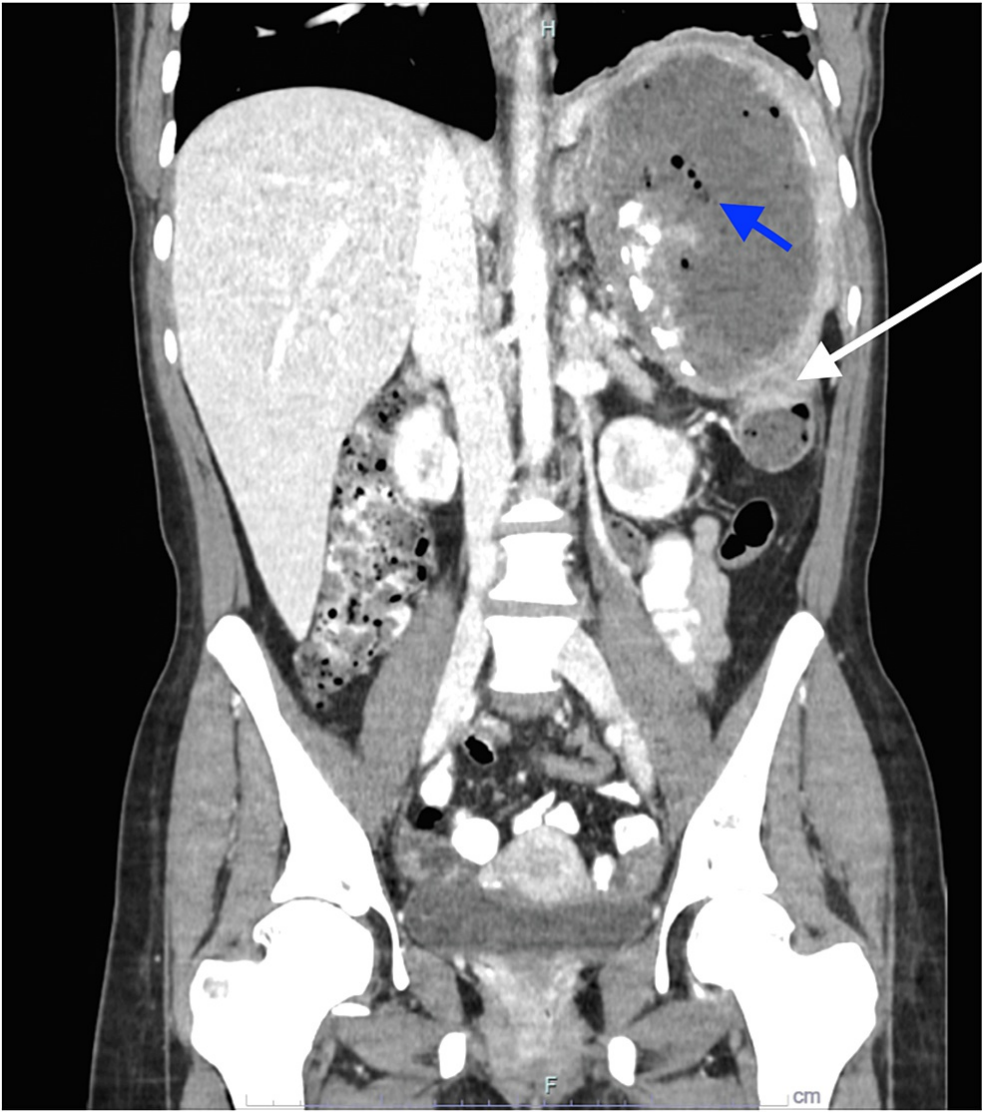
Coronal section of the CT scan of the abdomen showing the entire replacement of the spleen with abscess cavity, air locules in the cavity (blue arrow), and thickening of the peritoneal reflection (white arrow).

**Figure 3:**
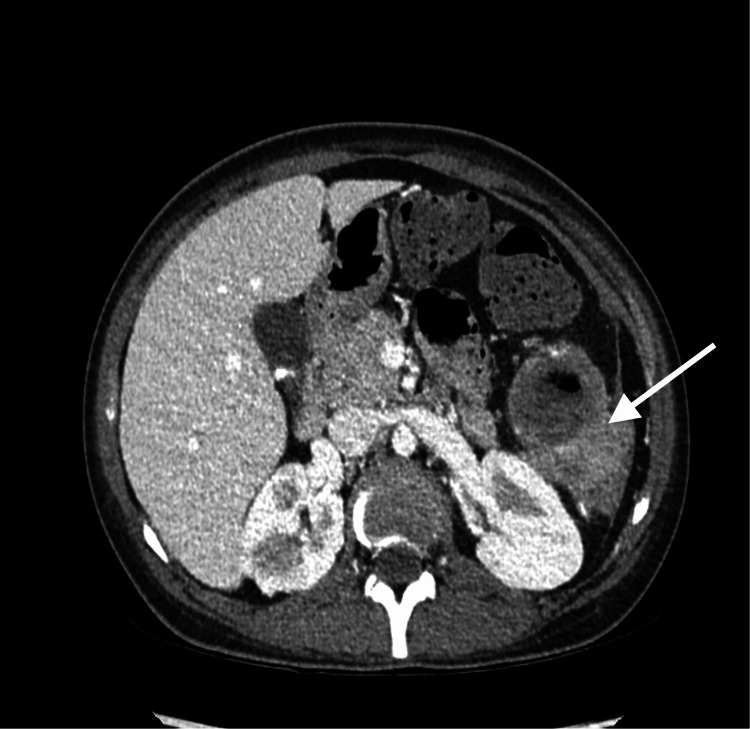
CT scan of the abdomen showing a mild thickening of the adjacent peritoneal reflection at the lower pole of the spleen (white arrow).

A CT scan disclosed a large cavitary lesion within the spleen with an air-fluid level indicative of post-embolization infarction and a large abscess collection (Figures 1, 2). There was a mild thickening of the adjacent peritoneal reflection suspicious for colosplenic fistula (Figures 2, 3). When we reviewed her previous images taken during her admissions for vaso-occlusive crises at our hospital, ultrasounds from 2014 and 2018 showed hepatomegaly, cholelithiasis, and an unchanged cystic lesion in the spleen, suggestive of necrosis or cystic degeneration. The spleen measured approximately 11 cm.

The patient was started on meropenem, vancomycin, and anidulafungin. Subsequent surgery revealed extensive adhesions involving the spleen, omentum, stomach, left lobe of the liver, and transverse colon. The significantly enlarged spleen displayed very thin, calcified parenchyma and an abscess that had nearly replaced the entire organ. Notably, a colosplenic fistula at the level of the splenic flexure was identified, necessitating conversion from laparoscopic to open surgery for abscess drainage, splenectomy, and segmental colonic resection with primary anastomosis.

Pathological examination of the excised large bowel tissue showed acute inflammatory changes and adhesions with perforation due to the splenic pathology. There was no evidence of inflammatory bowel disease, granulomas, microorganisms, dysplasia, or malignancy. The spleen pathology revealed extensive necrosis and infarction with cystic degeneration and secondary acute inflammation. No normal spleen tissue remained. Microbiology confirmed a splenic abscess caused by *Escherichia coli* and *Bacteroides* species. Postoperatively, the patient tolerated the procedure well and was discharged on day eight. The patient recovered well, was discharged home, and continued to do well on follow-up.

## Discussion

The development of a colosplenic fistula following a splenic abscess in this patient underscores a rare but serious complication of splenic embolization [6-11]. The literature suggests that splenic embolization can lead to complications such as abscess formation, with a reported incidence of up to 4.3% in cases of non-operative management of blunt splenic injuries [6]. Partial splenic embolization (PSE) is utilized to decrease the need for transfusions and improve hematologic parameters in patients with hypersplenism and SCD. In a prospective study, Bazeboso et al. included 35 patients with SCD and hypersplenism who underwent PSE. The most frequent complication was splenic rupture (4/35, 11.4%) [7]. According to another study by Ahuja et al., splenic infarcts not requiring any treatment occurred in 21% of patients, and 3% later developed a splenic abscess [8]. Such complications necessitate careful consideration in patients with pre-existing conditions such as SCD, where the risk of morbidity is heightened. None of these cases reported a colosplenic fistula, which may take a long time to develop into a fistula to nearby structures. In our case, the patient developed a colosplenic fistula a long time after the splenic embolization.

Pathological examination of the colon and the spleen in this case revealed acute inflammatory changes and perforation adjacent to the splenic lesion, without evidence of malignancy or inflammatory bowel disease. This finding is consistent with the literature, which reports various causes for colosplenic fistulae, including inflammatory diseases, trauma, and complications from previous medical interventions [12-19]. In a recent case series and literature review, the predominant etiologies were colonic lymphoma (30%) and colorectal carcinoma (17%) [19].

The more common signs and symptoms were fever (90%), chills (41%), and abdominal pain (31%). Splenic abscesses can be diagnosed with CT or ultrasound (US). A case series of 29 cases of splenic abscess showed a sensitivity of 100% for CT and 93% for US [20]. In the study by Hernandez et al., CT was commonly used for diagnosis (90%) [19]. However, diagnosing a colosplenic fistula remains a challenge. In our case, CT scans and ultrasound of the abdomen were used. The CT scan showed large cavitary lesions, with mild thickening of the adjacent peritoneal reflections, indicating a possible colosplenic fistula. The colosplenic fistula was identified intraoperatively, leading to a conversion to an open approach for splenectomy and colonic resection. Surgical management was successful with no operative complications.

Most colosplenic fistula cases necessitated surgical intervention. In the same series, about 87% of patients underwent surgery, predominantly involving segmental resection (81%) of the impacted colon and splenectomy (77%). Initially, 19 patients were managed surgically, and 12 were managed non-operatively. However, 11 of the non-operative patients eventually needed surgery due to unresolved symptoms [19].

This case adds to the body of evidence suggesting a need for a high index of suspicion for rare complications following splenic embolization.

## Conclusions

Colosplenic fistula is a rare long-term complication after splenic embolization. This case reinforces the need for vigilant monitoring and a high index of suspicion for potential complications following splenic embolization, especially in patients with chronic abdominal pain. The successful surgical management of this patient’s colosplenic fistula underscores the importance of timely intervention in achieving favorable outcomes.
